# Supplementary material for: Altered microRNA Transcriptome in Cultured Human Liver Cells upon Infection with Ebola Virus
Source: Int J Mol Sci. 2021 Apr 6;22(7):3792. doi: 10.3390/ijms22073792 (PMC8038836; doi:10.3390/ijms22073792)
Supplement: Supplementary file 1 [file ijms-22-03792-s001.zip › Supplementary_File/C_ GO_Analysis_Results/16-30nt_go_Reston-24h-Huh7_vs_Control-24h-Huh7_down.mature_mirna_targets/MF_FoldEnrichmentDotPlot.pdf]

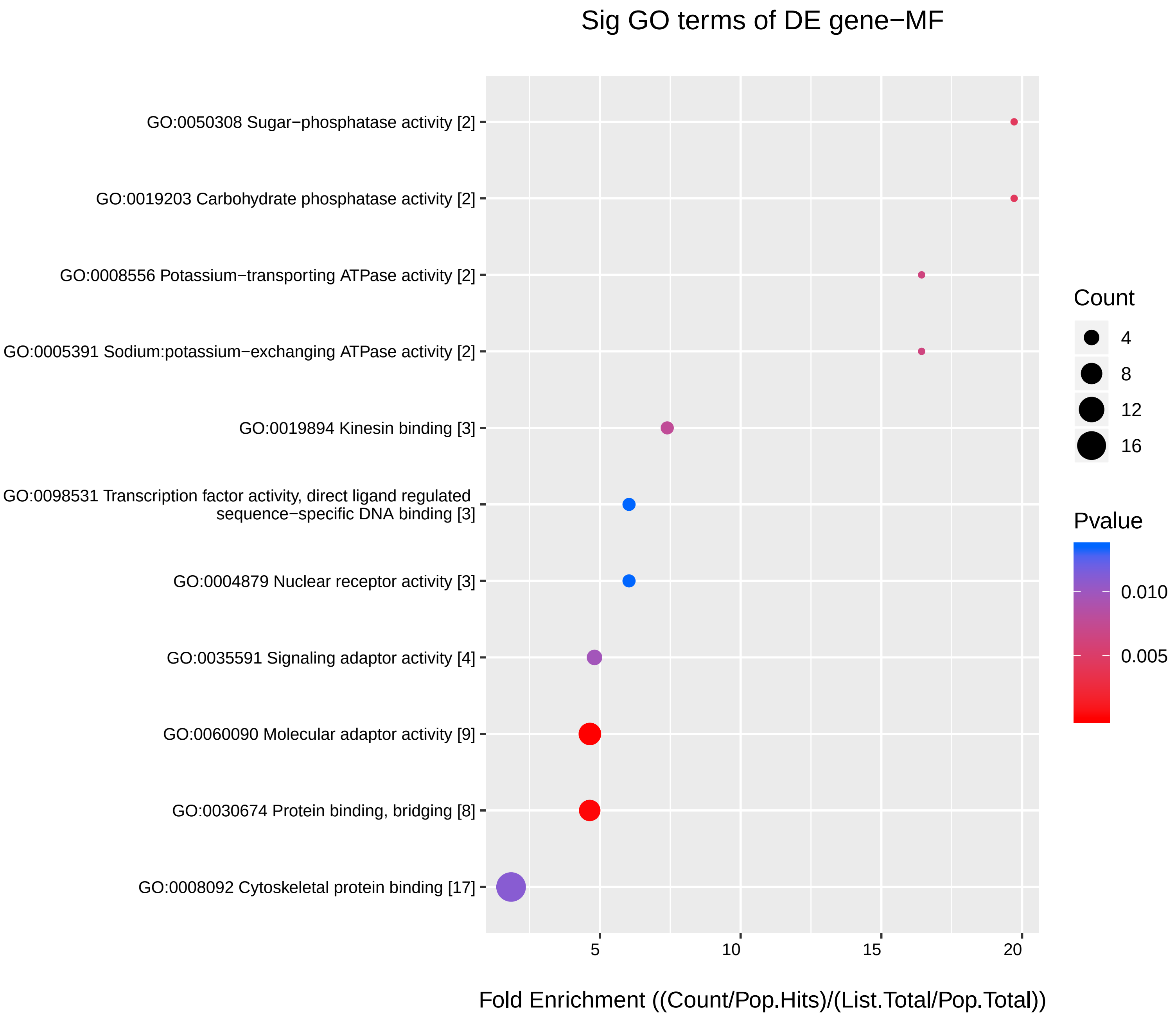

Sig GO terms of DE gene−MF
GO:0050308 Sugar−phosphatase activity [2]
GO:0019203 Carbohydrate phosphatase activity [2]
GO:0008556 Potassium−transporting ATPase activity [2]
GO:0005391 Sodium:potassium−exchanging ATPase activity [2]
GO:0019894 Kinesin binding [3]
GO:0098531 Transcription factor activity, direct ligand regulated sequence−specific DNA binding [3]
GO:0004879 Nuclear receptor activity [3]
GO:0035591 Signaling adaptor activity [4]
GO:0060090 Molecular adaptor activity [9]
GO:0030674 Protein binding, bridging [8]
GO:0008092 Cytoskeletal protein binding [17]
5
10
15
20
Fold Enrichment ((Count/Pop.Hits)/(List.Total/Pop.Total))
Count
4
8
12
16
Pvalue
0.010
0.005
